# High anti-tumor activity of a novel alpha-fetoprotein-maytansinoid conjugate targeting alpha-fetoprotein receptors in colorectal cancer xenograft model

**DOI:** 10.1186/s12935-023-02910-0

**Published:** 2023-04-05

**Authors:** Patricia Griffin, Wendy A. Hill, Fabio Rossi, Rebecca Boohaker, Karr Stinson, Igor Sherman

**Affiliations:** 1 Biocatalyst4Development Inc., 33 Markham Road, Scarborough, ON M1M 2Z5 Canada; 2 Abzena Ltd., Babraham Research Campus, Cambridge, CB22-3AT UK; 3 Southern Research Institute, 2000 Ninth Avenue South, Birmingham, AL 35205 USA; 4 Alpha Cancer Technologies Inc., MaRS Centre-South Tower, 200-101 College Street, Toronto, ON M5G 1L7 Canada

**Keywords:** Alpha-fetoprotein-maytansinoid conjugate, Colorectal cancer, AFP receptor, Tumor targeting

## Abstract

The alpha-fetoprotein receptor (AFPR) is a novel target for cancer therapeutics. It is expressed on most cancers and myeloid derived suppressor cells (MDSCs) but generally absent on normal tissues. Studies were performed to investigate the use of recombinant human AFP (ACT-101) conjugated with maytansinoid toxins for targeted toxin delivery to cancer. Four structurally different ACT-101-maytansinoid conjugates containing cleavable glutathione sensitive linkers were initially investigated in a mouse xenograft model of colorectal cancer. Reduction in tumor volume was seen for all four conjugates compared to control (p < 0.05). The anti-tumor effects of the conjugate selected for further development (ACT-903) persisted after treatment discontinuation, with tumors becoming undetectable in 9 of 10 mice, and all 10 mice surviving through Day 60 with no obvious signs of toxicity. A follow-up study performed in the same model compared the effects of single intravenous doses of ACT-903 (10–50 mg/kg) to that of control groups receiving vehicle or ACT-101. A significant reduction of tumor burden compared to control was achieved in the 40 and 50 mg/kg dose groups. Survival was significantly prolonged in these 2 groups (40 mg/kg (p < 0.0001); 50 mg/kg (p = 0.0037). Free maytansine blood levels at 4 h were 0.008% of the dose, indicating stability of the conjugate in circulation as was expected based on in vitro plasma stability studies. No obvious signs of toxicity were seen in any of the treated groups. Observed efficacy and excellent tolerability of ACT-903 in these xenograft models support advancing the development of ACT-903 toward clinical use.

## Introduction

The alpha fetoprotein receptor (AFPR) is expressed on the surface of many common cancers, including solid tumors such as breast, lung, ovarian, colorectal, prostate and hematologic tumors as well as on myeloid derived suppressor cells (MDSCs) [1] but generally absent on normal adult cells [22, 31, 33]. Targeting the AFPR for selective delivery of toxins to tumor cells has previously been proposed as an anticancer strategy [15, 18].

Alpha fetoprotein (AFP) is the natural ligand for the AFPR and is one of the most studied oncofetal antigens [23]. In the fetus, AFP functions as a shuttle protein, similar to albumin, bringing amino acids, fatty acids and other hydrophobic molecules required for cell growth and metabolism into the cell by reversibly binding to these molecules and then delivering them into the cell via the AFPR [6, 25]. Once inside the cell, AFP releases the nutrients into the cell and then returns to the circulation to resume shuttling additional molecules into cells [6]. AFP levels decrease abruptly after birth and are virtually undetectable in adult, although serum AFP levels are elevated in some patients with hepatocellular carcinoma and it is used as a clinical biomarker for diagnosis in this condition [32]. AFP could serve as an ideal protein for the targeted transport of toxin to cancer cells expressing the AFPR since AFP is selectively endocytosed by cancer cells but not by normal adult cells.

Evidence for existence of a specific receptor for AFP was first demonstrated on the MCF-7 human breast cancer cell line [27] and later on the U937 human lymphoma cell line [21]. Scatchard analysis in both studies was consistent with the presence of at least two binding sites for AFP of different affinities. Since then, specific binding of AFP to the AFPR has been demonstrated in a variety of human cancers or cancer cell lines, [11, 15, 17] consistent with the high AFPR expression observed on tumor cell lines including colorectal, ovarian, breast, lung and lymphoma in our own studies.

The exact structure of the AFPR is not fully elucidated, presumably due to its complex polymer configuration and carbohydrate composition, although recently, the mucin family and scavenger receptor family have been proposed to function as AFPRs [9, 10]. Nevertheless, specific tumor uptake of radiolabeled AFP via the AFPR has been demonstrated in vivo, in C3H/Bi mice which overexpress estrogen and aromatase and develop spontaneous mammary tumors [26], as well as in mice bearing various human prostate, breast and endometrial xenografts [11]. In addition, Moro reported experience with injection of ^131^I-AFP in three cancer patients showing clear accumulation of radioactivity corresponding to the palpable mass in a patient with stomach cancer, and to sites of metastasis in a patient with breast cancer. In contrast no radioactive spots were detectable in a breast cancer patient in complete remission [17].

ACT-101 is a non-glycosylated form of human AFP produced by recombinant DNA technology. It differs from naturally occurring AFP by one amino acid substitution (glutamine for asparagine at aa 233). We used ACT-101 as the targeting protein in the design of protein drug conjugates to deliver maytansinoid payloads to tumor cells. Maytansinoids, including DM1, DM3 and DM4, exert a potent anti-mitotic effect by suppressing microtubule dynamic instability [19] and their use in targeted antibody drug conjugates (ADCs) has been clinically validated. Kadcyla® (traztuzumab emtansine; Genentech/Roche), the first DM1-containing ADC on the market, was initially approved in 2013 by the FDA for the treatment of patients with HER2-positive, metastatic breast cancer. Kadcyla® contains a non-reducible thioether linker, with release of the cytotoxic catabolites of DM1 occurring following receptor-mediated internalization and subsequent lysosomal degradation. Numerous other ADCs containing maytansinoids are in development (clinicaltrials.gov). Mirvetuximab soravtansine (Immunogen), recently approved by FDA as monotherapy in patients with platinum-resistant ovarian cancer and high folate receptor–alpha expression, contains a cleavable disulfide linker which releases DM4 in the cytoplasm [16].

**Fig. 1:**
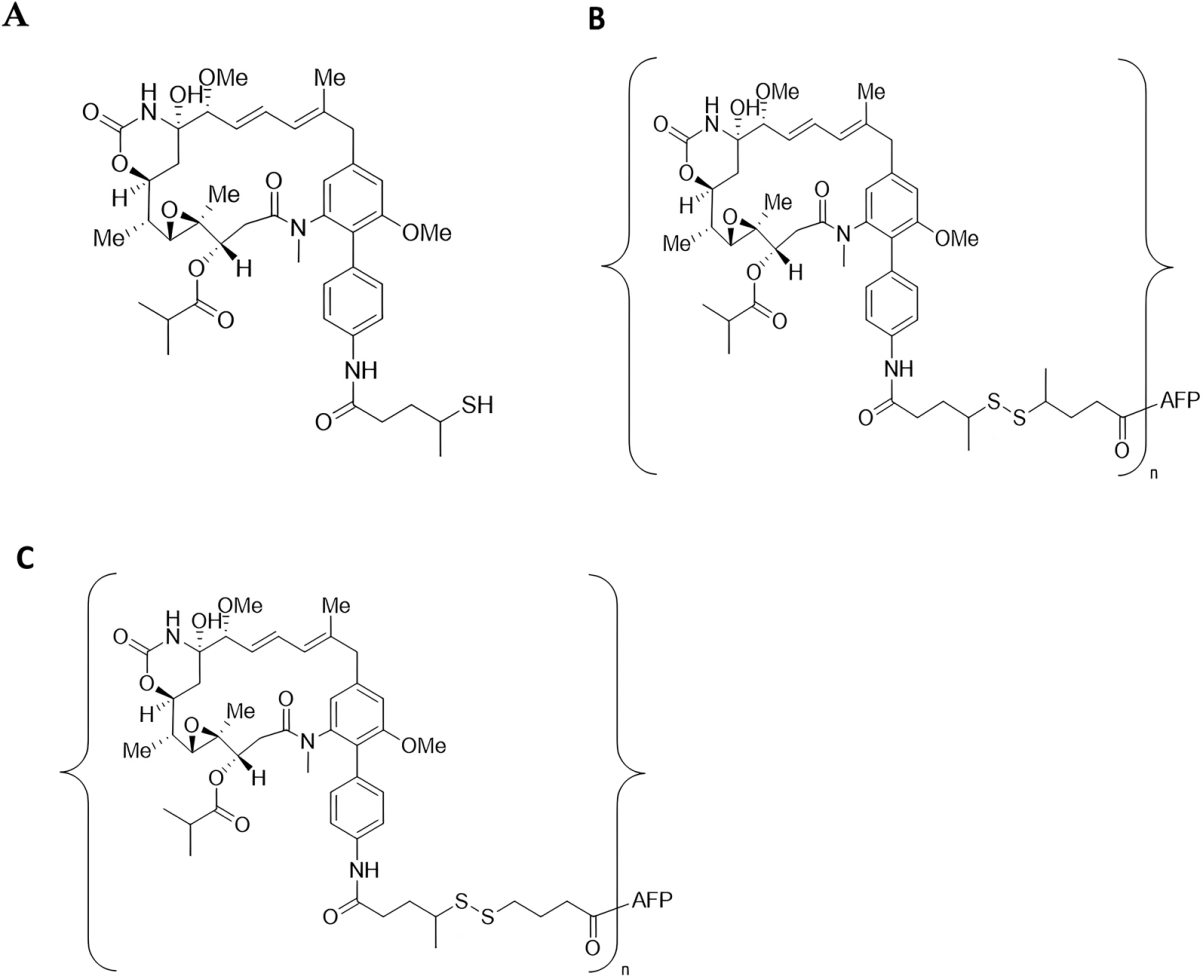
Structures of ABZ981 maytansinoid, ACT-101-ABZ982 and ACT-101-ABZ-1827 conjugates.** A** Chemical structure of ABZ981 maytansinoid; **B** Chemical structure of ACT-101-ABZ982 conjugates where the disulfide linkage is dimethylated **C** Chemical structure of ACT-101-ABZ1827 conjugates where the disulfide linkage is monomethylated. For **B** and **C** the toxin is coupled to ACT-101 via a primary amine and n is the number of toxin molecules per protein

The anti-tumor effects of ACT-101-maytansinoid conjugates incorporating DM1, DM4 and a proprietary maytansinoid (ABZ981, Fig. 1A) were compared in vitro in a panel of cell lines expressing the AFPR, including COLO-205. The biodistribution and efficacy of selected conjugates were further evaluated in vivo in mouse models of colorectal cancer.

## Materials and methods

### In vitro* characterization of AFPR expression in tumor cell lines*

Fluorescently labeled ACT-101 (F-ACT-101) was used to indirectly measure AFPR expression on a panel of cell lines, by evaluating the surface binding (conducted on ice) and binding/internalization (conducted at 37 °C). Binding experiments were performed using plate-based assays or by flow cytometry.

For binding/internalization experiments F-ACT-101 was prepared using Alexa Fluor™ 647 protein labeling kit obtained from Life Technologies. Following labelling, F-ACT-101 concentrations were determined using a Nanodrop spectroscopic assay and diluted with medium to provide 0.5 μM AFP for each tumor cell binding experiment. Cell lines studied included metastatic colorectal adenocarcinoma (COLO-205), ovarian adenocarcinoma (OVCAR-3, SKOV-3,), breast adenocarcinoma (MCF-7), non-small cell lung adenocarcinoma (H522), histiocytic lymphoma (U937) and acute lymphoblastic leukemia (MOLT-4). All cell lines were obtained from ATCC (passage numbers 2 to 9). In brief, tumor cells were cultured in a 100 mm dish (plated cells) or in an Eppendorf vial (suspension cells) and cell counts determined using Trypan Blue exclusion. Pilot experiments were performed to determine optimal cell density for suspension cells and plated cells at 2 to 4 million cells/100 μL. Cell lines were incubated with 0.5 μM F-ACT-101 at 37 °C for 0.5, 2 and 5 h (n = 3 replicates) and incubation was stopped on ice. Cells grown in suspension were collected by centrifugation and subjected to repeated wash steps. For plated cells, trypsin/EDTA was used for cell detachment as verified by microscopy followed by centrifugation to collect the cell pellet. The trypsin-containing medium was transferred back to wash the remaining cells in the dish. A 100 μL aliquot of all wash medium and cell pellets were sonicated for 10 min with 100 μL DMSO and then transferred to a 96 well plate for fluorescence measurement using a SpectraMax® microplate reader. Background fluorescence in vehicle control wells were subtracted and fluorescence due to F-ACT-101 remaining in cells was converted to ng/mL based on a calibration curve. Binding was expressed as ng F-ACT-101 per million cells.

Additional binding assays were performed by Southern Research, Birmingham, Alabama in the COLO-205 and U937 cell lines using F-ACT-101 prepared using the Alexa Fluor™ 488 Microscale Protein Labeling Kit (ThermoFisher Cat#A30006). Cells obtained from ATCC were cultured in RPMI-1640 media supplemented with 10% FBS at 37 ºC and 5% CO_2_ for at least two passages before use. The day before use media was changed to RPMI-1640 media supplemented with 2% FBS. Cells were harvested, washed and adjusted to 1 × 10^6^ viable cells/mL and incubated in serum-free RPMI-1640 on ice for 2 h. Cells were then washed and exposed to F-ACT-101 in cold PBS for 1 h on ice at concentrations of 1, 3, 10, 30 and 100 µg/mL. Following binding incubation, cells were washed with Cytometry Staining Buffer (CSB, BioLegend) and resuspended in 100 µL CSB. Cells were then analyzed on an Intellicyt iQue Screener Plus flow cytometer using Forcyt software for control of acquisition and analysis.

To demonstrate the specificity of binding of ACT-101 and ACT-101-maytansinoid conjugates a competitive binding assay was employed at Abzena Ltd. (Cambridge, UK) whereby U937 cells were transferred to fetal bovine serum-free medium for 2 h at 37 ºC. Cells were then co-incubated with F-ACT-101 at 25 µg/mL and increasing concentrations of unlabeled ACT-101 or ACT-101 conjugates in PBS + 0.1% NaN3 for 1 h at 4 ºC. Cells were then washed, fixed and fluorescence measured using an Attune NxT flow cytometer.

### Preparation of ACT-101-maytansinoid conjugates

ACT-101-maytansinoid conjugates were prepared by Abzena Ltd. (Cambridge, UK). Purity and drug-to-protein ratio (DPR) was analyzed by SEC-UV and LC–MS and protein concentration by Bradford Assay. The initial series of conjugates synthesized for in vitro analysis in a tumor cell line panel incorporated the maytansinoid payloads DM1, DM3, DM4 or ABZ981 at a drug to protein ratio (DPR) of 4.4 to 7.5. The three conjugates tested in the in vivo biodistribution study incorporated the payloads DM1, DM4 or ABZ981 at a DPR of approximately 4.0. The four conjugates tested in the multiple dose COLO-205 efficacy study all incorporated ABZ981 but differed in the DPR (approximately 4 or 6) and in the distribution of methyl groups around the disulfide linkage. Conjugates containing a di-methylated disulfide linker conjugates were designated ACT-101-ABZ982 (Fig. 1B), while conjugates containing a mono-methylated disulfide linker were designated ACT-101-ABZ1827 (Fig. 1C) and further characterized by the average DPR. The ACT-101-ABZ1827 conjugate with a DPR of about 5.96 was designated as ACT-903.

### In vitro* cytotoxicity testing*

For in vitro cytotoxicity studies conducted at Abzena, Cambridge, UK, U937 cells (passage 9) were seeded in 96 well plates at a density of 1.5 × 10^3^/well in 50 μL medium After 24 h, growth medium was replaced by serial dilutions of conjugates in medium over an 8-point concentration range. Following 96 h of incubation in the presence of conjugates, viability was detected using the CellTiterGlo® Luminescent Cell viability Assay.

In vitro potency of conjugates in COLO-205 cells was by assessed by Southern Research, Birmingham, Alabama. COLO-205 cells were grown in RPMI-1640 media supplemented with L-glutamine and 10% fetal bovine serum and seeded onto 96 well plates at either at 5,000 or 2,500 cells per well in a total volume to 50 μL per well. Plates were incubated overnight then treated with the conjugate at 37 °C for 72 h. An 8-point dilution series of 2X stocks of each conjugate in media were prepared and 50 μL of these stocks were added to cells to give final concentrations of 0.19 – 25 nM for ACT-101-ABZ1827 high DPR and 0.39 – 50 nM for ACT-101-ABZ982 high DPR and ABZ981 (toxin-linker) control. Samples were run in triplicate in two separate runs. Following 72 h of incubation in the presence of conjugates, viability was detected using the CellTiterGlo® Luminescent Cell viability Assay (Promega, Madison, WI, USA, Cat#G750). Luminescence was recorded on a Synergy 4.0. The conjugate wells were normalized to cell only controls and ABZ981 was normalized to DMSO treated controls. 4PL fit was performed in Graph Prism 8.2.1 and the IC50s determined.

### In vivo* studies*

The following in vivo experiments were conducted at Southern Research, Birmingham, Alabama USA. Animals were cared for in compliance with the Standard Operating Procedures of Southern Research.

For all studies NCr-*nu/nu* mice were obtained from Charles River. For studies in tumor-bearing mice, COLO-205 cells were received as NCI number 0507319, RRID: CVCL_0218, source authentication: ATCC, and were frozen down at Southern Research at passage 13 and at passage 21 specifically for use in these studies. All cells were screened for genetic drift prior to use. For xenograft studies, ten million (1 × 10^7^) cells were subcutaneously (SC) implanted on the right flank of 6–8 week-old mice on Day 0 and allowed to grow to measurable tumors over 14 days.

Initially, a biodistribution study of 3 ACT-101-maytansinoid conjugates: ACT-101-DM1, ACT-101-DM4 and ACT-101-ABZ982 at DPR of approximately 4.0 was performed. A single intravenous dose (25 mg/kg) of each conjugate was administered to COLO-205 tumor-bearing mice via intravenous (IV) tail injection. Animals were observed once daily from treatment to necropsy for morbidity and mortality. Body weights (bw) and tumor measurements were collected the day before treatment began. Concentration in blood, tissues and tumor was assessed by measuring both ACT-101 levels and free maytansine plus metabolites at 4, 8 and 24 h post dose (n = 3 per timepoint). ACT-101 serum levels were measured using commercial kits purchased from ALPCO (Salem, NH) according to the manufacturer’s instructions. For measurement of maytansine plus metabolite levels, plasma and tissue samples were spiked with a proprietary maytansinoid (Abzena Ltd) as the internal standard (IS). Analytes and IS were extracted in ethyl acetate:methanol (following homogenization of tissues), centrifuged and dried using an Eppendorf 5301 centrifugal evaporator. Calibration curves were prepared for each maytansine and metabolite using serial dilutions of standard solutions spiked with the proprietary maytansinoid IS. Reconstituted samples and calibration standards solutions were analyzed by LC–MS/MS on an Acquity UPLC in line with a Xevo G2S QTOF using a 15 min gradient elution with 0.1% formic acid in acetonitrile. MS/MS analysis was performed in positive ion mode, and the response of a specific fragment for each analyte was used for quantification. Concentrations were calculated from the analyte-specific calibration curves corrected for recovery based on the proprietary maytansinoid.

In a follow-up study, the maximum tolerated dose (MTD) of four conjugates, all of which released ABZ981 as the free toxin (Fig. 1A), but with different linker-toxin configurations and DPRs was determined in non-tumor bearing mice prior to testing these conjugates in a xenograft study. Group1: ACT-101-ABZ982 (DPR 3.74), Group 2: ACT-101-ABZ982 (DPR 5.81), Group 3: ACT-101-ABZ1827 (DPR 3.92), Group 4: ACT-101-ABZ1827 (DPR 5.96), Group 5: Vehicle control (n = 12/group). Each conjugate was administered at a dose of either 5,10, 20 or 40 mg/kg/day for a total of 10 doses (n = 3 per dose group for a total of 12 mice per conjugate). Each mouse was treated once a day by IV tail injection for 5 consecutive days, with 2 days off and then for a further 5 days. Daily clinical observations (mortality and morbidity and changes in body weight (bw) were used to assess for toxicity. All animals were euthanized 24 h after the final dose of conjugate at which time liver enzyme measurements were taken and livers were weighed and assessed for any visible lesions. Finally, livers were placed in 10% neutral buffered formalin and then processed to paraffin blocks for further histopathology evaluation.

An in vivo efficacy study was then conducted using the doses determined from the MTD study for each conjugate. Group numbers are the same as above. Groups 1 and 4 were dosed at 20 mg/kg and Groups 2 and 3 were dosed at 10 mg/kg.) Fifty (50) mice were randomized on the day prior to treatment (n = 10 per group). Treatment was initiated when tumors reached 100 to 200 mm^3^ (mean tumor volume of 150 mm^3^), on day 8 after implantation. Clinical signs (mortality, morbidity and toxicity) were assessed daily. Tumor measurements and body weight were assessed on the day prior to treatment, daily during treatment and twice weekly thereafter for 60 days following implantation. Tumor response was calculated as the % difference at each time point between mean tumor weight in each treatment group and the mean tumor weight of the control group (Group 5) at the same time point. All surviving animals were euthanized at day 60 post implantation.

ACT-101-ABZ1827 (DPR 5.96), subsequently designated as ACT-903, was tested in a separate efficacy study as a single IV injection at different doses in the same xenograft model. Treatment was initiated when the tumors reached 100 to 200 mm^3^ in size (mean volume of approximately 150 mm^3^) by IV tail injection on Day 9. Each dose group (N = 10) was administered at either 10, 20, 30, 40 or 50 mg/kg of ACT-903 or 25 mg/kg of ACT-101 (targeting protein only) or vehicle control by IV tail injection 9 days after tumor implantation. A second dose was administered to animals in the 40 and 50 mg/kg of ACT-903 on Day 24 post tumor implantation. Efficacy was assessed as in the study above. In addition, for animals surviving to day 60 post implantation, liver, spleen, heart and tumor were removed for macroscopic review for abnormalities.

## Results

### AFPR is expressed on a variety of human tumor cell lines

In the panel of human tumor cell lines, increasing binding/internalization of F-ACT-101 (in terms of ng protein/million cells) over the 5-h incubation period at 37 °C was observed for all cell lines except MOLT-4 and SKOV-3. At 5 h, MCF-7 (breast cancer) displayed the highest AFPR levels, followed by U937, COLO-205, H522, OVCAR-3 and SKOV-3. The binding of F-ACT-101 was estimated by subtracting background fluorescence in vehicle control wells from fluorescence due to F-ACT-101 remaining in cells after washing and was converted to ng/mL based on a calibration curve. Binding was expressed as ng F-ACT-101 per million cells. (Table 1). Due to the relatively low fluorescence response observed for MOLT-4 cells at 5 h, the calculated concentrations were below the lower limit of quantitation (LLOQ) for the assay, suggesting that the AFPR is low or absent from this cell line.

**Table 1:** Binding of F-ACT-101 to a panel of tumor cell lines (ng/million cells)

Time (hr)	MOLT-4	U937	COLO-205	H522	MCF-7	OVCAR-3	SKOV-3
0	0.0	0.0	0.0	0.0	0.0	0.0	0.0
0.5	8.1	29.5	6.9	55.6	51.0	67.0	31.3
2	19.0	67.1	33.6	27.1	53.6	21.2	9.2
5	LLOQ*	84.3	75.0	71.3	126.0	71.0	16.0

*Results below LLOQ for the assay

**Fig. 2:**
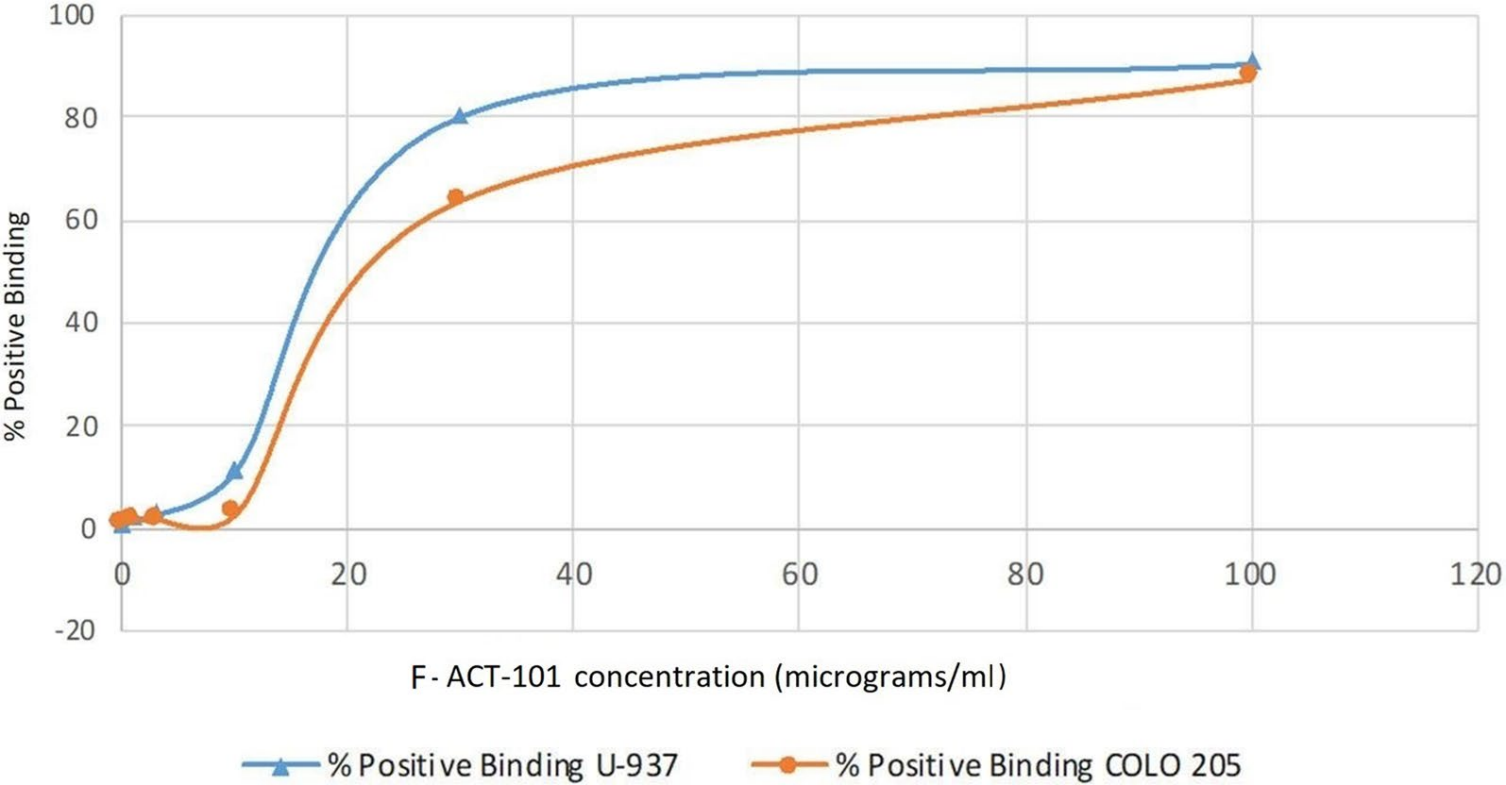
Concentration dependent binding of F-ACT-101 to COLO-205 and U937 cells. U937 or COLO-205 cells were exposed to F-ACT-101 in cold PBS for 1 h on ice at concentrations of 1, 3, 10, 30 and 100 µg/mL. Fluorescence was measured using flow cytometry. Maximum binding of F-ACT-101 for both cell lines (~ 90%) was observed at 100 µg/mL

Results of binding studies of ACT-101 to COLO-205 and U937 cell lines performed on ice are shown in Fig. 2. Binding of F-ACT-101 to COLO-205 and U937 showed a similar concentration-dependence, with a maximum of close to 90% of cells positive for F-ACT-101- binding.

### AFP Conjugates demonstrate cytotoxicity against tumor cells in vitro

Cytotoxicity of ACT-101 conjugates containing the maytansinoid toxins DM4 (ACT-101-DM4), DM1 (ACT-101-DM1) or ABZ981 (ACT-101-ABZ982) prepared at a DPR of approximately 4 were examined in vitro. All conjugates were cytotoxic against the U937 and COLO-205 cell lines with the DM1 and DM3-containing conjugates being more potent in vitro than ACT-101-ABZ982 (Table 2). The U937 cell line was more sensitive than the COLO-205 cell line to all conjugates, possibly due to a higher level of expression of the AFP receptor.

**Table 2:** In vitro cytotoxicity of ACT-101 Conjugates with different maytansinoid toxins

Sample	DPR	U-937		COLO-205	
		n	AVG ± SD (nM)	n	AVG ± SD (nM)
ACT-101-DM4	4.1	2	3.20 ± 0.64	2	8.51 + 0.98
ACT-101DM1	4.0	3	6.09 ± 0.13	3	7.77 ± 3.47
ACT-101-ABZ982	4.0	3	13.52 ± 2.45	3	38.58 ± 4.30
DM4	N/A	2	2.22 ± 0.41	2	1.29 ± 0.68
DM1	N/A	3	2.74 ± 0.72	3	5.42 ± 0.59
ABZ981	N/A	3	3.28 ± 0.60	3	3.92 ± 0.26

*DPR* Drug to Protein Ratio; DM4, DM1, ABZ981—maytansinoids alone; AVG ± SD: Average concentration in nM ± Standard Deviation

### In vivo delivery of toxin to tumor highest with ACT-101-ABZ982 conjugate

Prior to administration of conjugates in vivo, an ex vivo study was performed which showed all conjugates to be stable after incubation in mouse or human serum for 7 days at 37 °C (data not shown, on file with Alpha Cancer Technologies Inc.). Delivery of toxin to COLO-205 tumors implanted in NCr-nu/nu mice after a single IV injection was higher with the ACT-101-ABZ982 conjugate, compared to the ACT-101-DM4 and ACT-101-DM1 conjugates, with uptake evident at 4 h post-dose and peak concentration at the 8-h timepoint (Table 3A).

**Table 3:** Tumor and plasma levels of toxin by conjugate

Time (hr)	DM1 conjugate		DM4 conjugate		ABZ981 conjugate	
	Free DM1 ng/mg tissue	% of total dose administered*	Free DM4 ng/mg tissue	% of total dose administered*	Free ABZ981 ng/mg tissue	% of total dose administered*
A. Tumor level						
4	0.0137 ± 0.0084	0.013 ± 0.002%	0.0040 ± 0.0014	0.003 ± 0.001%	0.0766 ± 0.0047	0.068 ± 0.030%
8	0.0647 ± 0.0222	0.075 ± 0.058%	0.0041 ± 0.0003	0.003 ± 0.001%	0.1860 ± 0.0834	0.266 ± 0.213%
24	nd	n/a	0.0086 ± 0.0038	0.007 ± 0.001%	0.0658 ± 0.0753	0.030 ± 0.029%
B. Plasma levels						
0.25	0.019 ± 0.0081	0.0012 ± 0.0005	0.003 ± 0.0001	0.0002 ± 0	0.006 ± 0.006	0.0004 ± 0.0004
1	0.009 ± 0.003	0.0006 ± 0.0002	0.002 ± 0.0002	0.0001 ± 0	0.003 ± 0.002	0.0002 ± 0.0001
4	0.020 ± 0.007	0.0013 ± 0.0004	n/a	n/a	0.006 ± 0.0004	0.0004 ± 0
8	0.017 ± 0.0013	0.0011 ± 0.00001	n/a	n/a	0.006 ± 0.002	0.0003 ± 0.0001
24	0.003 ± 0.001	0.0002 ± 0.00001	n/a	n/a	0.002 ± 0	0.0001 ± 0

*n/a* Not available as average of non-detected or below LOQ data points

*Calculated as: $$\% {\text{ of total dose administered }} = \frac{{{\text{measured concentration }}\left( {{\text{ng}}/{\text{mg}}} \right) \, \times {\text{ estimated tissue weight }}\left( {{\text{mg}}} \right) \times { 1}00\% }}{{{\text{Amount of drug injected based on body weight }}\left( {{\text{ng}}} \right)}}$$

**Fig. 3:**
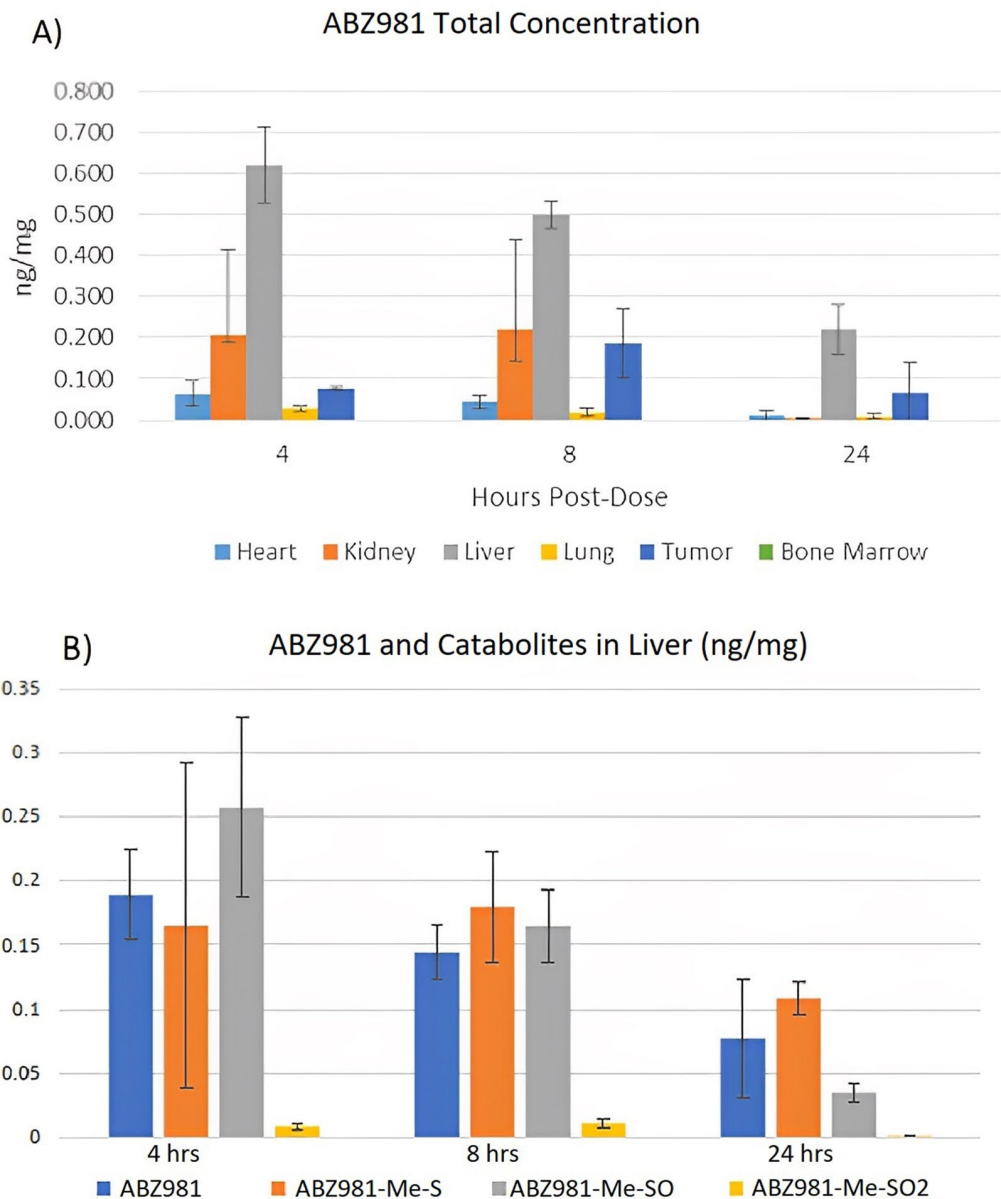
Biodistribution and metabolism of the ACT-101-ABZ982 DPR 4.0 conjugate. **A** Toxin levels (free ABZ981 and metabolites) concentration (ng/mg) in homogenized tumor and off target tissues (heart, kidney, liver, lung and bone marrow) at 4, 8 and 24 h after drug administration, detected using LC–MS/MS (n = 3 per timepoint); **B** Free toxin (ABZ981) and metabolites: S-methyl derivative (ABZ981-Me-S), S-methyl-sulfoxide derivate (ABZ981-Me-SO) and S-methyl sulfone derivative (ABZ981-Me-SO2) concentration (ng/mg) in homogenized mouse liver tissue at 4, 8 and 24 h after drug administration (n = 3 per timepoint)

Concentration in the tumor and off-target tissues with the ACT-101-ABZ982 conjugate is shown in Fig. 3A. There were no detectable toxin levels in the bone marrow over the 24-h observation period with any of the conjugates. Peak toxin levels for the ACT-101-ABZ982 conjugate in heart and lung were detectable but low, only 0.03% of the total dose administered and decreasing with time, but higher in kidney and liver, ~ 0.36% and ~ 2.5% of the total dose, respectively, consistent with elimination pathway of maytansine in vivo. Analysis of metabolites in liver homogenates using LC–MS/MS revealed S-methylation of the free maytansinoid and conversion to the corresponding S-methyl sulfoxide and S-methyl-sulfone. All metabolites decreased over the 24 h time period, as clearly shown for the ACT-101-ABZ982 conjugate in Fig. 3B.

All conjugates showed good stability in blood with negligible plasma levels of free toxin detected (Table 3B). For the ACT-101-ABZ982 conjugate, mean peak levels were 0.006 ng/mL, representing only 0.0004% of the total dose administered. In light of this, ACT-101 levels were used to represent intact conjugate levels in blood. The serum levels of ACT-101 over time were similar with all conjugates, and consistent with the expected half-life of the protein in tumor-bearing mice of approximately 7 h (data on file at Alpha Cancer Technologies Inc.) indicating that the half-life in circulation of these conjugates is primarily driven by the protein.

### ACT-101-ABZ981 conjugates with different linkers and different DPRs exhibit specific tumor cell binding and cytotoxicity

**Fig. 4:**
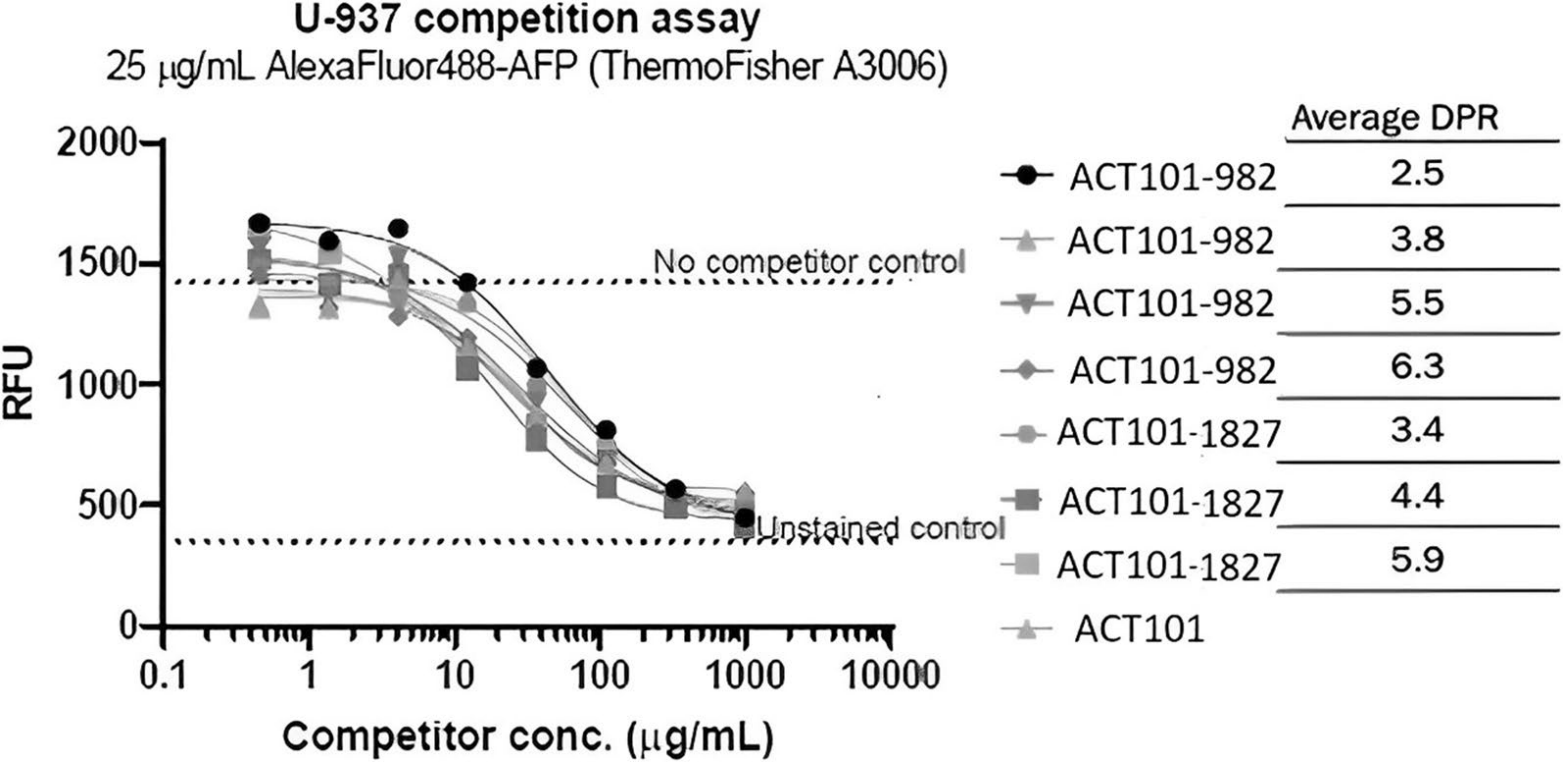
ACT-101 conjugates with different linkers and DPRs show specific binding to U937 tumor cells. U937 cells were co-incubated with F-ACT-101 at 25 µg/mL and with increasing concentrations of each conjugate or ACT-101 for 1 h at 4 ºC. Fluorescence was measured by flow cytometry. All conjugates showed similar specificity of binding to U937 cells as the unconjugated form of the protein (ACT-101)

Based on the results of the biodistribution study described above, conjugates containing the proprietary maytansinoid, ABZ981, but with two differing linkers, a di-methylated disulfide linker ACT-101-ABZ982 (Fig. 1B) or a mono-methylated disulfide linker conjugates ACT-101-ABZ1827 (Fig. 1C), were selected for further investigation and synthesized at a range of DPRs. All conjugates prepared at DPRs of 2.5 to 6.3 were shown to compete with ACT-101 for binding to U937 tumor cells, with similar or overlapping competition curves (Fig. 4). These results confirm the specificity of binding of conjugates within this range of DPR.

Bioconjugates with the higher DPR were more potent than the lower DPR bioconjugates in vitro, with the exception of ACT-101-ABZ982 DPR 6.3. However the IC50 value for this conjugate was based on a single experiment (Table 4).

**Table 4:** Cytotoxicity of ACT-101 conjugates with different linkers and DPRs in U937 cells

Conjugate	Average DPR	IC50** (nM) (n = 2)
ACT-101-ABZ982	2.5	5.25
ACT-101-ABZ982	3.8	5.29
ACT-101-ABZ982	5.5	1.71
ACT-101-ABZ982	6.3	6.55***
ACT-101-ABZ1827	3.4	7.07
ACT-101-ABZ1827	4.4	5.98
ACT-101-ABZ1827	5.9	2.93
Free Toxin (ABZ-981)		3.06

*DPR* Drug to Protein Ratio; **IC50: the concentration of drug required for 50% growth inhibition; ***n = 1

Based on the results, 2 conjugates for each of the release functionalities were prepared at DPRs of approximately 4 (“low DPR”) and 6 (“high DPR”) for testing in an in vivo colorectal xenograft tumor model. Prior to in vivo testing, cytotoxicity against COLO-205 cells in vitro was confirmed with the high DPR conjugates (Table 5). Both conjugates demonstrated similar comparable nM potency to the ABZ981 linker-payload. Results were similar at both cell seeding densities used.

**Table 5:** Cytotoxicity (IC50) of high DPR ACT-101-ABZ982 and ACT-101 ABZ1827 conjugates in COLO-205 cells

	5000 cells/well (Plate 1)	5000 cells/well (Plate 2)	2500 cells/well (Plate 1)	2500 cells/well (Plate 2)
ACT-101-ABZ982 high DPR	4.32 nM	5.14 nM	3.17 nM	3.60 nM
ACT-101-ABZ1827 high DPR	6.52 nM	7.13 nM	5.08 nM	4.88 nM
ABZ981(linker-payload)	6.86 nM	7.29 nM	4.92 nM	5.17 nM

Cell viability was detected using the CellTiterGlo® Luminescent Cell Viability Assay

### ACT-101 conjugates show efficacy in COLO-205 tumor bearing mice

The dose of each of the 4 conjugates used in the efficacy study described below was based on the results of the MTD study (Table 6). The MTD was determined to be below 40 mg/kg/day when administered by IV tail injection for 10 days (total dose of 400 mg/kg) for all conjugates. Two conjugates exhibited toxicities at doses of 20 mg/kg/day (elevated liver enzymes, bw decline) and therefore for the subsequent efficacy study the 10 mg/kg/day dose was selected for Groups 2 and 3 while the 20 mg/kg/day dose was selected for Groups 1 and 4, with the same regimen (5 consecutive days, with 2 days off and then 5 more days) being used for all groups. A further gross pathology examination of the livers found no visible lesions on any of the livers in the conjugate or vehicle control groups. However, microscopic evaluation of the livers from the highest of dose group of each conjugate (40 mg/kg/day dose) found signs of hepatoxicity (hepatocyte hypertrophy and necrosis) when compared to the control livers. This further supported the selection of doses below 40 mg/kg/day for the efficacy study.

**Table 6:** Maximum Tolerated Dose (MTD) determination for dose selection for efficacy study

Group No	1	2	3	4
Conjugate	ACT-101-ABZ982 low DPR	ACT-101-ABZ982 high DPR	ACT-101-ABZ1827 low DPR	ACT-101-ABZ1827 high DPR
DPR	3.74	5.81	3.92	5.96
0 mg/kg (n = 3)	No findings	No findings	No findings	No findings
5 mg/kg (n = 3)	No findings	No findings	No findings	No findings
10 mg/kg (n = 3)	No findings	↓bodyweight, ↑AST & ALT (not significant) (MTD)	Slight hemolysis (MTD)	No findings
20 mg/kg (n = 3)	Slight hemolysis ↑AST & ALT (NS) (MTD)	↓bodyweight, dehydration, ↓liver weight, ↑AST & ALT	↑AST & ALT	No findings (MTD)
40 mg/kg (n = 3)	↓bodyweight, dehydration, ↓liver weights, ↑AST & ALT	Deaths, ↓bodyweight, rash, ↓liver weight, ↑AST & ALT	↓bodyweight ↑AST & ALT	Deaths, ↓bodyweight, ↓liver weight, ↑AST & ALT

*AST* aspartate transaminase, *ALT* alanine aminotransferase, *DPR* drug to protein ratio, *mg* milligrams, *kg* kilograms, *NS* not significant, *MTD* maximum tolerated dose; ↑: increased; ↓: decreased

**Fig. 5:**
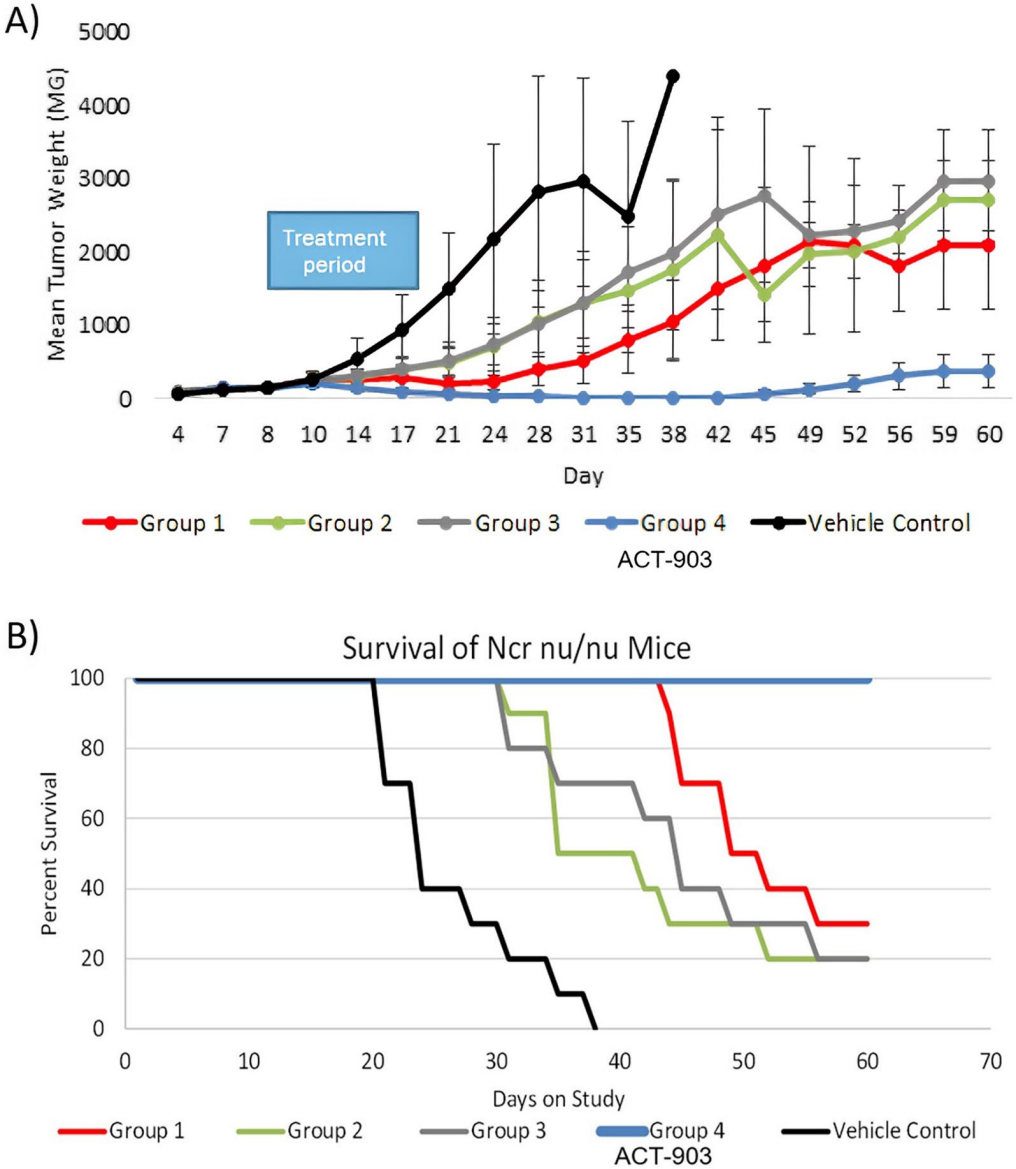
Efficacy of 4 ACT-101-maytansinoid conjugates in the COLO-205 xenograft model. **A** Inhibition of tumor growth. Changes in mean tumor weight following implantation (tumor weight is estimated as V = (L xW^2^)/2 where V is volume (with density assumed to be close to 1 g/mL and Weight = V), L is tumor length, and W is tumor width; The bars represent standard deviation, n = 10 per group. Statistical significance was determined by Kruskal Wallace & Dunnett’s on Ranks, followed by Wilcoxon test. Tumor growth was significantly slower (p < 0.05) in all groups compared to vehicle control. **B** Survival. Calculated as % of animals alive on a given day compared to total number of animals in the group. All surviving animals were euthanized at day 60 post implantation. Group 1: ACT-101-ABZ982 (DPR 3.74) 20 mg/kg, Group 2: ACT-101-ABZ982 (DPR 5.81) 10 mg/kg, Group 3: ACT-101-ABZ1827 (DPR 3.92) 10 mg/kg, Group 4: ACT-101-ABZ1827 (DPR 5.96) 20 mg/kg, Group 5: Vehicle control

As shown in Fig. 5A, tumor growth was slower in all conjugate groups compared to control with a significant reduction (p < 0.05) by Day 14 which continued through Day 24, five days after completion of treatment. In Group 4 tumor regression continued post-treatment, with no tumors palpable in 9 of 10 mice by Day 38. In this group, tumor regression was statistically different compared to Groups 2 and 3 on Day 24 (p < 0.0001) and compared to Group 1 on Day 35 (p = 0.001).

With respect to survival, all animals in Group 4 were alive through to Day 60 with only minimal tumor growth during the last week of the study (Fig. 5B) compared to control where all animals were dead due to excessive tumor growth by Day 38. There were no treatment-related toxicities or deaths observed in this study. Based on the results of this efficacy study, the Group 4 ACT-101-ABZ1827 conjugate with an average DPR of 5.9 was selected for further development and designated as ACT-903.

**Fig. 6:**
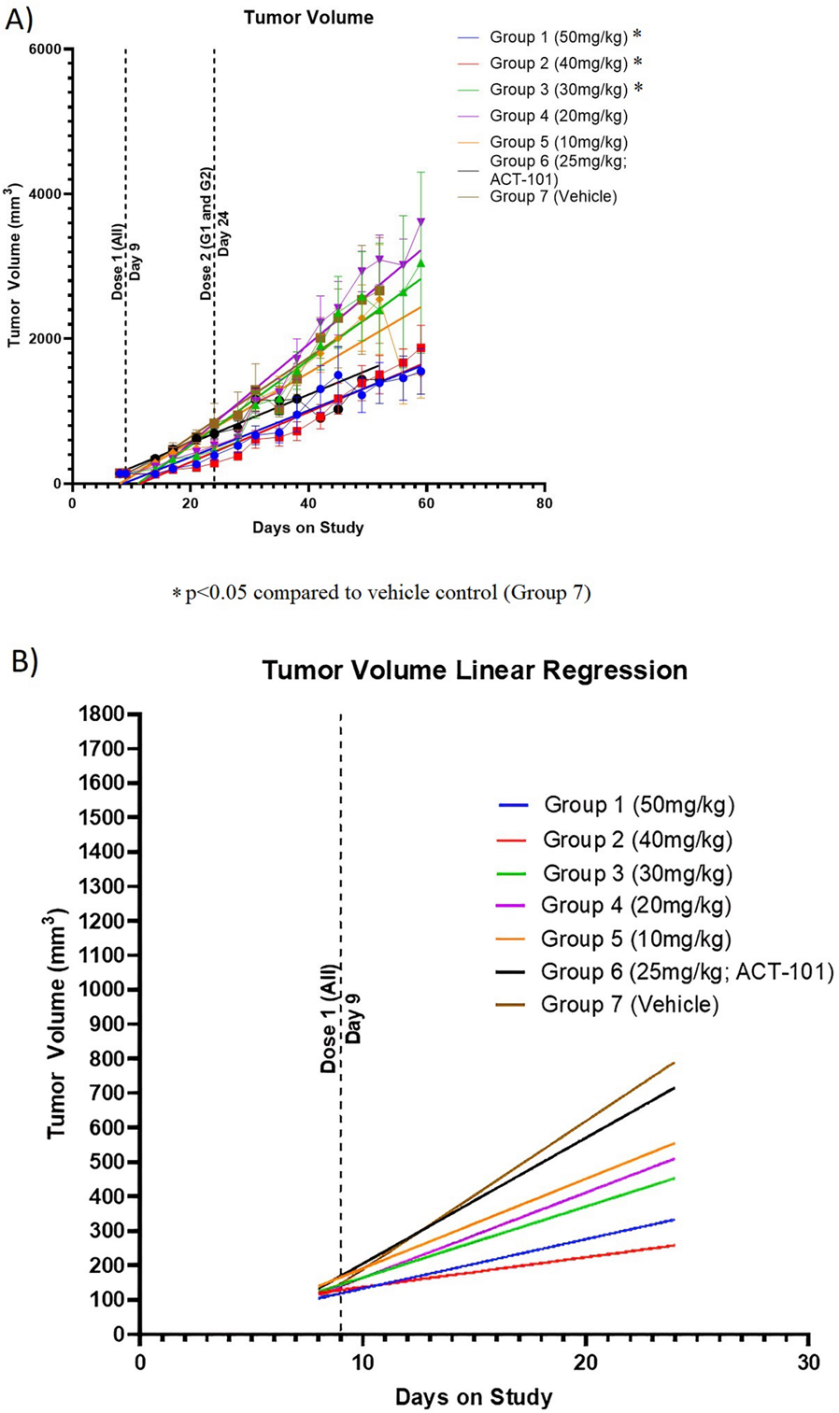
Dose-dependent effects of ACT-903 on tumor volume.** A** Tumor volume. Each dose group (n = 10 per group) was administered either 10, 20, 30, 40 or 50 mg/kg of ACT-903 or 25 mg/kg of ACT-101 (targeting protein only) or vehicle control by IV tail injection. Dose 1 was administered to all animals on Day 9. A second dose (Dose 2) was administered to animals in the 40 and 50 mg/kg groups (G1 and G2) on Day 24 post tumor implantation. Statistical significance was determined by Kruskal Wallace & Dunnett’s on Ranks, followed by Wilcoxon test. **B** Tumor volume linear regression. Effect of first dose on tumor growth kinetics using linear regression analysis of the tumor volumes over time between the first and second doses

In the single dose study investigating a range of doses of ACT-903 (n = 10/group), there was a statistically significant reduction in tumor volume compared to Group 7 (control) or Group 6 (ACT-101 control) by Day 14 in the 50 mg/kg (Group 1), 40 (Group 2) and 30 mg/kg (Group 3) groups (p < 0.05) (Fig. 6A). Group 4 and Group 5, 20 mg/kg and 10 mg/kg, respectively, were not significantly different from the 2 control groups. The largest percent reduction in tumor volume from control was recorded on Day 14 for Group 1 (– 58%) and on Day 24 for Group 2 (– 65.9%). A second dose administered on Day 24 to Groups 1 (50 mg/kg) and 2 (40 mg/kg) served to maintain the percent reduction in tumor volume relative to control at approximately – 50% for the remainder of the study. For Group 3 (30 mg/kg) the largest percent difference in tumor volume was observed on Day 14 (– 35.9% relative to vehicle; p < 0.05). An examination of the slope of the line between the first dose and second dose of ACT-903 shows a dose-dependent inverse correlation between the dose and slope of the linear regression (Fig. 6B). The rate of tumor growth in a group administered ACT-101 alone was somewhat slower when compared to vehicle.

**Fig. 7:**
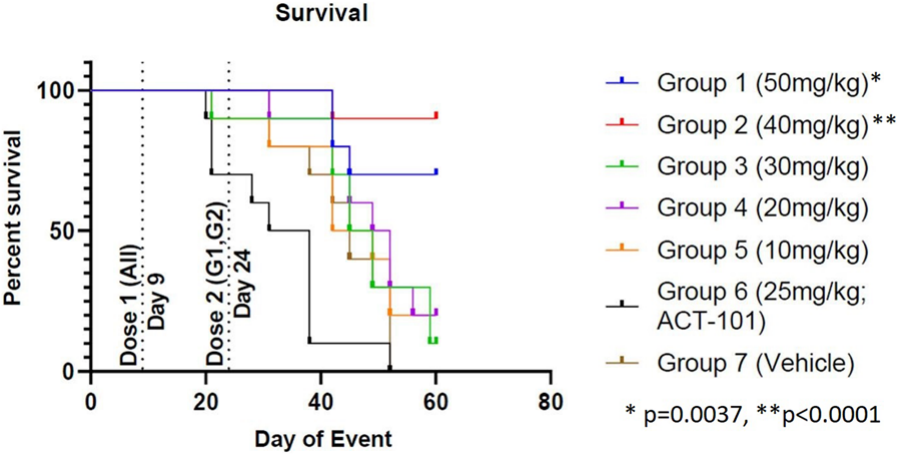
Dose-dependent effects of ACT-903 on survival

Survival was significantly prolonged in the 40 mg/kg (p < 0.0001) and 50 mg/kg (p = 0.0037) dose groups (Groups 1 and 2). Survival in the ACT-101 protein control group was worse (p = 0.0386) than the vehicle control group (Fig. 7).

Dose 1 was administered to all animals on Day 9. A second dose (Dose 2) was administered to animals in the 40 and 50 mg/kg groups (G1 and G2) on Day 24 post tumor implantation. %survival is calculated as % of animals alive on a given day compared to total number of animals in the group. All surviving animals were euthanized at day 60 post implantation. Statistical comparisons relative to Group 7 (control) were performed using Log Rank (Mantel-Cox) test.

Free maytansine levels in plasma at 4 h post dose represented 0.008% of the injected dose, indicating stability of the conjugate in circulation. There were no significant differences in body weights between groups and the average body weight remained consistent for the duration of the study. Necropsy on liver, heart and spleen did not reveal any visible lesions or abnormalities in any animal. No ocular toxicity or tail scarring were observed in any of the dosing groups.

## Discussion

**Fig. 8:**
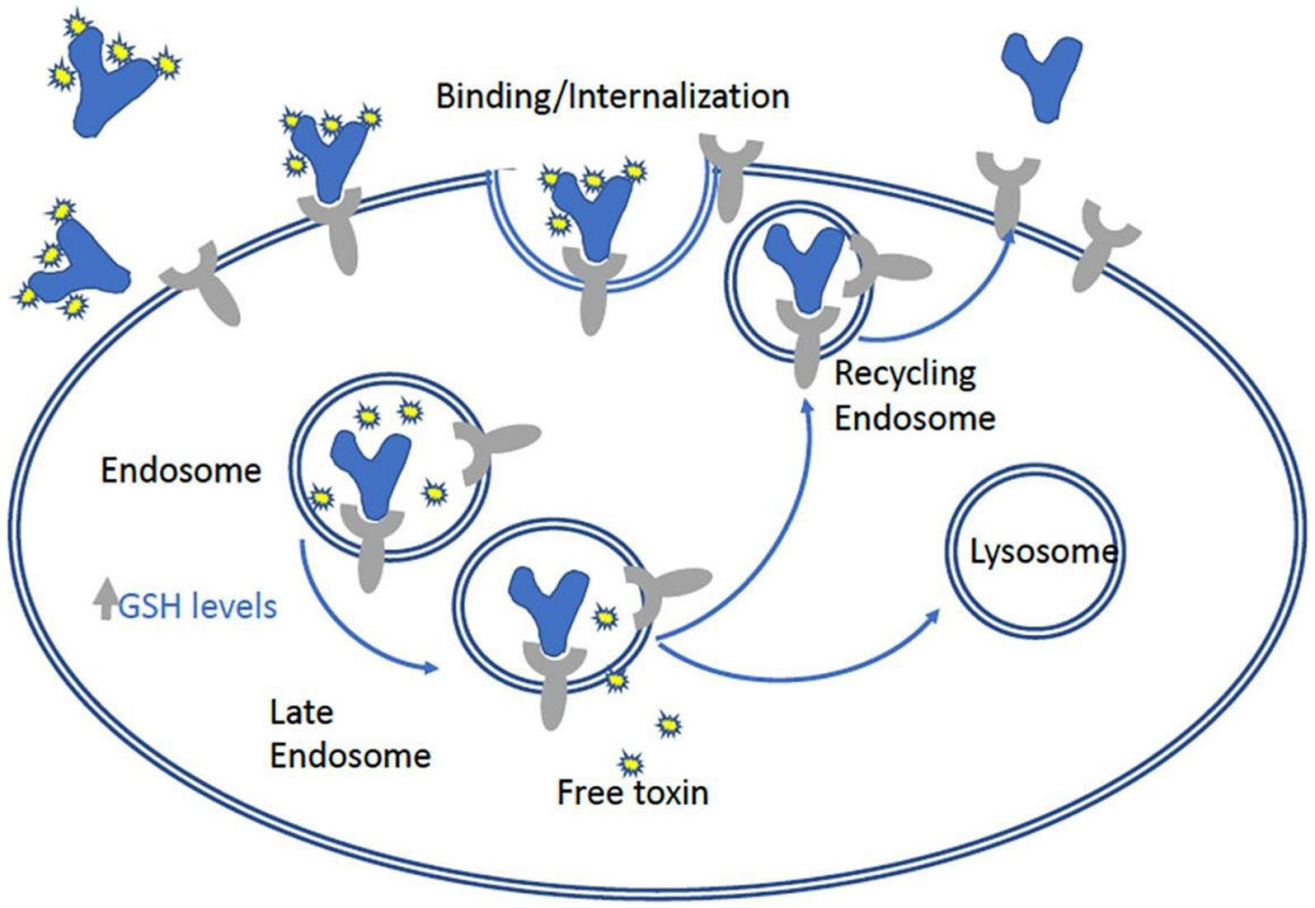
Dual tumor targeting mechanism of ACT-101 conjugates. Once ACT-101 conjugate (blue Y shape with toxins attached (small yellow star shapes) binds to the AFP receptor (grey Y shape), the conjugate is endocytosed and transported to the interior of the cell, where high glutathione (GSH) concentrations result in linker cleavage and release of the cytotoxic payload. Some of the ACT-101 may be transported outside the cell by recycling endosomes

AFP internalization by cancer cells through the AFPR is associated with enhanced growth and malignant behaviour of tumors [9, 10], consistent with the observation of modestly increased tumor growth in unconjugated ACT-101 group in this study. An attempt to block AFP uptake using antibodies against the AFPR was reported by Moro [17] but it was not very effective in reducing tumor growth. ACT-101-maytansinoid conjugates represent a more effective strategy in that they take advantage of the natural function of AFP as a carrier protein to deliver a toxic payload to the cancer cell (Fig. 8). Furthermore, because ACT-101 is a human protein to which every fetus is exposed, the risk of immune reactions or development of anti-drug antibodies is low. In Phase II studies of ACT-101 (formerly MM-093) in rheumatoid arthritis conducted by Merrimack Pharmaceuticals In., no infusion reactions or other immune reactions to ACT-101 were observed (data on file at Alpha Cancer Therapeutics Inc.).

There are reports of conjugates utilizing other forms of AFP which have demonstrated anti-tumor effects both in vitro and in vivo. However, these conjugates incorporated less potent payloads and utilized different linker strategies. These included conjugates which were non-covalently associated with daunomycin conjugated to fatty acids [2] and paclitaxel-loaded nanoparticles attached to a fragment of AFP [7]. Conjugates prepared using glutaraldehyde as crosslinking agent inhibited tumor growth and improved survival in mice inoculated with B16 melanoma [4]. Direct conjugation of doxorubicin with a fragment of AFP using an acid labile linkage [31] resulted in conjugates with high toxicity towards doxorubicin-sensitive SKOV3 cells as well as doxorubicin-resistant SLVB ovarian cancer cells while showing low toxicity to normal lymphocytes compared to doxorubicin.

For ACT-101-maytansinoid conjugates, a glutathione sensitive linker was selected for attaching the toxin to the protein. Glutathione is present in relatively high concentrations inside the cells compared to blood and is even higher in cells experiencing stress or hypoxic conditions, and therefore is present in higher concentrations in cancer cells than in blood or in normal cells [8, 12]. Although other release mechanisms were considered (e.g., pH and proteases-sensitive linkages), the glutathione release mechanism is most relevant for ACT-101-cytotoxin conjugates, since AFP does not traffic to lysosomes where protease-labile linkers can be cleaved. Glutathione-sensitive disulfide linkers are one of the most attractive strategies for cleavable linkers in ADC design since the disulfide bond increases the stability of the linker in circulation which reduces the likelihood of off-target release/toxicity [8, 14]. This strategy has been clinically validated, with two ADCs employing glutathione sensitive disulfide linker already approved by the FDA and a number in advanced clinical development, including other maytansinoid based antibody–drug-conjugates [28]. ACT-101-maytansinoid conjugates provide a dual tumor-targeting mechanism based on both differential expression of the AFPR on tumors and increased glutathione concentration in tumor cells (Fig. 8).

The selection of maytansinoid as the toxin was based on its proven efficacy in the clinic when incorporated into ADCs. The toxicity profile of maytansinoid-ADCs appears to correlate with the stability of the conjugates in the bloodstream and the extent of the off-target effects [5]. In clinical trials of ADCs incorporating maytansinoids, bone marrow toxicity was the most frequent adverse event of grade 3 or greater. The very low percentage of free toxin detectable in blood after administration of the ACT-101-maytansinoid conjugates in the biodistribution study, as well as the lack of toxicity observed in the efficacy studies are consistent with the expected blood stability of conjugates incorporating a disulfide linker. Furthermore, the lack of any bone marrow accumulation of maytansine or its metabolites observed with all of the ACT-101-maytansinoid conjugates tested in the biodistribution study is a positive finding suggesting reduced potential for bone marrow toxicity in the clinic.

The metabolites detected are consistent with the reported hepatobiliary route for maytansinoid elimination [3], where the free maytansinoids are first S-methylated and ultimately eliminated through the liver by oxidation to the corresponding S-methyl sulfoxide and S-methyl-sulfone metabolites. Oxidized metabolites of DM1 and DM4 have been shown to have relatively low cytotoxic potency in cell based assays [3, 29].

Tumor targeting was better with the ACT-101-ABZ982 conjugate, than with the DM-based conjugates when prepared at a similar DPR (approximately 4.0), with tumor levels observed in the biodistribution study of approximately 3 and 22 times higher than with the DM1 and DM4 conjugates respectively. The best efficacy in the COLO-205 xenograft model was observed with the ACT-101-ABZ1827 DPR 5.9 conjugate (ACT-903). This is likely related to the higher DPR and to a lesser degree of steric hindrance around the disulfide bond.

Colorectal cancer is the third most commonly diagnosed cancer in the United States and the second most common cause of cancer-related death, accounting for 9.4% of all cancer-related deaths [30]. Chemotherapy with 5-FU plus oxaliplatin (FOLFOX) or leucovorin, 5-FU and irinotecan (FOLFIRI) is the standard of care for metastatic colorectal cancer (mCRC) with a median overall survival of approximately 18 to 20 months. Targeted biological therapies approved for mCRC include angiogenesis inhibitors such as bevacizumab and therapies targeting the EGF receptor (cetuximab and panitumumab) which are typically given in combination with chemotherapy. Immune checkpoint inhibitors (pembrolizumab and nivolumab) are approved for subsequent-line treatment of patients but are restricted to use in the 3 to 7% of patients with mismatch repair proteins/microsatellite instability. BRAF mutations are found in 8 to 12% of mCRC cases and BRAF kinase inhibitors are being investigated in combination with other therapies. HER-2 inhibition is also being investigated as amplification of this target is seen in 2 to 11% of mCRC cases [20]. In contrast, the AFPR is expected to be present in a much higher percentage of patients with colorectal cancer.

The dramatic, anti-tumor effects of ACT-903 in the absence any obvious signs of toxicity support further development of ACT-903 for the treatment of cancer, particularly metastatic colorectal cancer where there is a significant unmet medical need. In addition, the consistent finding of the expression of the AFPR across multiple tumor cell lines supports further in vivo investigations of ACT-903 across a wider range of tumor models in order to identify additional tumor types for clinical development. Finally, it is worth mentioning that efficacy studies described here were performed in immunocompromised mice and therefore only show the direct tumor targeting of ACT-903. However, AFPRs are also found on MDSCs present in the tumor microenvironment [13]. It is now well recognized that these MDSCs protect the tumor from immune surveillance [24] and attack. ACT-903 has the potential to target MDSC's in addition to cancer cells, leading to enhanced efficacy.

## Data Availability

Study data are available as an electronic copy in an online repository at Southern Research Institute.
